# Nudix Hydrolase as a Target for Regulating High Temperature Tolerance in Marine Diatoms

**DOI:** 10.3390/microorganisms14081625

**Published:** 2026-07-25

**Authors:** Ruihao Zhang, Yongliang Liu, Qi An, Baohua Zhu, Tengsheng Qiao, Yuhao Sun, Sen Wang, Kehou Pan, Jun Li, Xiaojin Song

**Affiliations:** 1 Laoshan Laboratory, Qingdao 266237, China; 2 Key Laboratory of Mariculture, Ocean University of China, Ministry of Education, Qingdao 266003, China

**Keywords:** Nudix hydrolases, diatoms, high temperature, stress tolerance, photosynthetic electron transport

## Abstract

Diatoms, as a vital component of marine ecosystems, face severe threats to their productivity and species composition due to increasing ocean temperatures. Although the effects of high temperatures on diatom growth and physiology have been thoroughly studied, the regulatory mechanisms in response to high temperature are still poorly understood. In this study, we identified Nudix hydrolases (Nudix) from the marine model diatom *Phaeodactylum tricornutum* as a key regulator of high temperature tolerance. We discovered that *Nudix* is a temperature-sensitive gene and its expression is induced by high-temperature. Under high-temperature stress, overexpression of *Nudix* markedly reduces reactive oxygen species (ROS) production and enhances ROS scavenging capacity, thereby decreasing lipid peroxidation and cell death rate to improve the high-temperature tolerance of *P. tricornutum*. Conversely, the silencing of *Nudix* induces excessive ROS production in *P. tricornutum* and reduces its high-temperature tolerance. Subcellular localization combined with the photosynthetic activity analyses further demonstrated that Nudix, localized in the chloroplast, prevents energetic electron accumulation and excessive reduction in the photosynthetic electron transport chain by increasing the electron transport rate, thereby suppressing ROS production at high-temperature stress. Collectively, our findings highlight the importance of Nudix in high-temperature tolerance of diatoms and provide insights into how marine diatoms acclimate to high temperature.

## 1. Introduction

Ocean warming caused by global climate change has become one of the most serious threats to marine ecosystems [1,2]. Diatoms, as a critical component of marine ecosystems, contribute approximately 20% of global primary productivity and nearly 25% of oxygen, playing a vital role in the marine food web and biogeochemical cycles [3,4]. Studies indicate that marine diatoms are extremely sensitive to high temperatures, which grow slowly and even stagnate at high temperature [5,6]. Consequently, ocean warming can significantly affect diatom productivity and species composition. Although the effects of temperature on diatom growth and physiology are well-studied [7,8], the molecular mechanisms underlying their response to high-temperature environments remain poorly understood.

Recently, a large number of studies have provided important insights into the high-temperature-tolerance molecular mechanisms of diatoms through genomics, transcriptomics, and proteomics [9,10,11]. However, existing studies have primarily focused on omics data, the functional studies of key temperature-responsive genes remain limited. As is well known, diatom heat tolerance is a complex biological process mediated by the synergistic interaction of multiple genes and pathways, which maintains survival at high temperature by regulating processes such as protein homeostasis, nutrient uptake, and the synthesis of polyunsaturated fatty acids [6,12,13]. Therefore, identifying and analyzing temperature-responsive genes is key to investigating the molecular mechanisms underlying diatom thermal tolerance.

Notably, previous studies discovered that Nudix hydrolases (Nudix), which are widely found in eukaryotes, play important regulatory roles in their response to stress [14,15,16]. Under multiple stress conditions, Nudix induces the expression of relevant stress-resistant genes, thereby enhancing stress tolerance [17,18,19]. However, research on Nudix primarily focuses on land plants, and its role in diatoms, particularly in response to high-temperature stress, has not yet been reported. Interestingly, our research group in previous marine diatom *Phaeodactylum tricornutum* study obtained two genetically engineered algal strains with significant differences in high-temperature tolerance [20] and found that the expression level of Nudix gene is positively correlated with high-temperature tolerance (Figure S1). The above findings remind us that Nudix may be involved in the high-temperature response of *P. tricornutum*. Considering the potential functions of Nudix identified in our previous studies, this study addressed the question of whether Nudix functioned in marine diatoms’ tolerance to high-temperature stress.

In this study, we intend to use the marine model diatom *P. tricornutum* as our subject of study. By combining genetic, biochemical and physiological approaches, we aim to elucidate the key role of Nudix in diatom high-temperature tolerance and its underlying mechanisms, thereby providing new insights for studying the regulatory mechanisms by which marine diatoms respond to high temperature.

## 2. Materials and Methods

### 2.1. Strains and Growth Conditions

Wild-type (WT) *P. tricornutum* (CCMP 2561) was provided by the Institute of Hydrobiology, Chinese Academy of Sciences. WT and genetically engineered (*Nudix* overexpression and silencing) strains were cultured in sterile f/2 medium under 100 µmol photons·m^−2^·s^−1^ with a 12 h:12 h light/dark cycle. During the experiment, the temperatures were set to 22 °C, 24 °C, 26 °C, 28 °C, 30 °C, respectively. All cultures were inoculated into 500 mL conical flasks containing 300 mL of culture medium, with the initial inoculation density adjusted to 1 × 10^6^ cells mL^−1^. All experiments were performed using biological triplicates, with the three replicates conducted independently.

### 2.2. Analysis of Subcellular Localization in Nudix

The enhanced green fluorescent protein (eGFP) gene was cloned to the 3′-end of *Nudix* and then inserted into the pPha-T1 vector to construct a fusion expression vector of Nudix and eGFP. The pPha-T1 vector containing only eGFP was used as the positive control vector. The above vectors were introduced into algal cells, and the fluorescence signals were observed using a laser scanning confocal microscope (Leica, Wetzlar, Germany). Notably, the excitation and emission wavelengths of eGFP were 488 nm and 520–540 nm; the excitation and emission wavelengths of chloroplast autofluorescence were 488 nm and 630–700 nm.

### 2.3. Construction of Nudix Overexpression and Silencing Strains

To construct the *Nudix* overexpression strains, we primarily synthesized the full-length coding sequences of *Nudix* and inserted it into the pPha-T1 vector, thereby obtaining the pPha-T1-OENudix overexpression vector. Additionally, antisense vector was constructed for gene silencing. For the antisense vector construction, the antisense fragment of Nudix was amplified via polymerase chain reaction (PCR) using specific primers, and this fragment was inserted into the pPha-T1 vector in the antisense orientation to construct the pPha-T1-ASNudix silencing vector. Notably, all of the above vectors contain a fragment of the bleomycin-resistant gene (*sh ble*).

The constructed *Nudix* overexpression and silencing vectors were transformed into *P. tricornutum* according to the method described by Zaslavskaia et al. [21]. Briefly, the constructed vectors were wrapped with tungsten particles, and the tungsten particles were introduced into the algae cells using the Bio-Rad Biolistic PDS-1000/He Particle Delivery System (Bio-Rad Laboratories, Hercules, CA, USA). The *Nudix* overexpression and silencing strains were screened on the f/2 solid medium (containing 1% agar) supplemented with 100 µg·mL^−1^ Zeocin™. Finally, the integration of the expression vectors into the genomes of the genetically engineered algal strains and the expression levels of *Nudix* were analyzed using PCR and quantitative reverse transcription polymerase chain reaction (qPCR) to ensure the successful overexpression and silencing of *Nudix*. All primers used in this section are listed in Supplementary Table S1.

### 2.4. Gene Expression Levels Analysis Using qPCR

First, total RNA was extracted and purified from algal cells using the Total RNA Kit I (Omega Bio-Tek, Norcross, GA, USA). In brief, 30 mL of algal cells was collected at 4 °C and 4000× *g* for 5 min using a Centrifuge 5804 R and Rotor F-34-6-38 (Eppendorf, Hamburg, Germany) as well as ground using liquid nitrogen. After grinding, the algal cells were thoroughly mixed with 1 mL of RNA-solv Reagent. After standing at room temperature for 2 min, 200 µL chloroform was added to mix, and the supernatant was obtained by centrifugation at 4 °C and 10,000× *g* for 15 min. A total of 540 µL of supernatant and 180 µL of anhydrous ethanol were added to the HiBind RNA column for mixing, and the waste liquid was discarded after centrifugation. Then, 400 µL Buffer I and 500 µL Buffer II were added respectively, and the waste liquid was discarded after centrifugation. Finally, 30 µL DEPC water was added to the RNA column, placed at room temperature for 2 min, and centrifuged at 4 °C and 10,000× *g* for 5 min to obtain RNA. Then, the RNA was reverse transcribed into cDNA and analyzed using the Applied Biosystems (Foster City, CA, USA) QuantStudio 5 Real-Time PCR System. The histone H4 gene was used as an internal control, and relative gene expression levels were calculated using the 2^−ΔΔCt^ method [20]. Detailed information on all primers used in the qPCR analysis is provided in Supplementary Table S1.

### 2.5. Detection of Stress Tolerance Characteristics

For the detection of cell death, SYTOX Green (1 µM, Invitrogen, Carlsbad, CA, USA) was used to identify dead cells. Briefly, the algal cells were collected by centrifugation at room temperature and 4000× *g* for 5 min using a Centrifuge 5804 R (Eppendorf, Germany), and 500 µL of f/2 medium was added to resuspend the cells after discarding the supernatant. Subsequently, 5 µL of SYTOX Green was added to achieve a final concentration of 1 µM, and the mixture was incubated for 30 min at 25 °C in the dark. After two washes, signal detection was performed using a flow cytometer CytoFLEX A00-1-1102 (Beckman, Brea, CA, USA) with an excitation wavelength of 488 nm and an emission wavelength of 520 nm. For each sample, 10,000 cellular events were collected. Algal cells inactivated with hydrogen peroxide during the assay were used as positive control. Unstained WT algal cells were used as negative controls to define the fluorescence threshold. Gating strategy was shown in Figure S2. Cell mortality was determined by calculating the percentage of SYTOX-positive cells relative to the total number of microalgal cells.

For the detection of ROS, the production of ROS in algal cells was measured using 2′,7′-dichlorodihydrofluorescein diacetate (DCFH-DA, Sigma-Aldrich, Saint Louis, MO, USA). DCFH-DA is hydrolyzed into DCFH upon entering algal cells; intracellular ROS oxidize DCFH to form the fluorescent compound DCF, and the level of intracellular ROS can be assessed by measuring the fluorescence intensity of DCF. Briefly, the algal cells were collected by centrifugation at room temperature and 4000× *g* for 5 min using a Centrifuge 5804 R, and 500 µL of f/2 medium was added to resuspend the cells after discarding the supernatant. Next, 5 µL of DCFH-DA (500 µM) was added to achieve a final working concentration of 5 µM and allow the reaction to proceed for 30 min at 25 °C in the dark. After washing twice, the algal cells were analyzed by flow cytometry using an excitation wavelength of 488 nm and an emission wavelength of 520 nm. The flow cytometer model, manufacturer, and acquisition settings were the same as those used for cell death rate detection. Finally, ROS production was determined by calculating the percentage of DCFH-positive cells among the total number of microalgal cells.

Malondialdehyde (MDA) content, superoxide dismutase (SOD), ascorbate peroxidase (APX), glutathione S-transferase (GST) and catalase (CAT) activities were measure using the MDA content assay kit, SOD activity assay kit, APX activity assay kit, GST activity assay kit and CAT activity assay kit (Solarbio, Beijing, China), respectively. Under acidic conditions and high temperature, MDA can condense with thiobarbituric acid to form a brownish-red compound, 3,5,5-Trimethyloxazolidine-2,4-dione, which has a maximum absorption wavelength of 532 nm. The MDA content in a sample can be estimated through colorimetric analysis. Briefly, the algae cells were collected by centrifugation, and the extraction buffer in the kit was provided for ultrasonic crushing (power 200 W, work 3 s, interval 5 s, repeat 30 times). After centrifugation, the supernatant was immediately detected by a microplate reader (Synergy HT, BioTek, Winooski, VT, USA) at 532 nm.

Superoxide anion (O_2_^−^) was produced by xanthine and xanthine oxidase reaction system. O_2_^−^ could react with WST-1 to form water-soluble yellow formazan, which was absorbed at 450 nm. SOD can scavenge O_2_^−^, thereby inhibiting the formation of formazan; the deeper the yellow color of the reaction solution, the lower the activity of SOD, and the higher the activity. Briefly, the algae cells were collected by centrifugation, and the extraction buffer in the kit was provided for ultrasonic crushing (power 200 W, work 3 s, interval 5 s, repeat 30 times). After centrifugation, the supernatant was immediately detected by a microplate reader at 450 nm.

APX catalyzes the oxidation of ascorbic acid (AsA) by H_2_O_2_, and the APX activity is calculated by measuring the oxidation rate of AsA. Briefly, the algae cells were collected by centrifugation, and the extraction buffer in the kit was provided for ultrasonic crushing (power 200 W, work 3 s, interval 5 s, repeat 30 times). After centrifugation, the supernatant was immediately detected by a microplate reader at 290 nm.

GST catalyzes the combination of Glutathione and 1-Chloro-2,4-dinitrobenzene, and the light absorption peak wavelength of the combined product is 340 nm. The GST activity can be calculated by measuring the increase rate of absorbance at 340 nm. Briefly, the algae cells were collected by centrifugation, and the extraction buffer in the kit was provided for ultrasonic crushing (power 300 W, work 3 s, interval 7 s, total time 3 min). After centrifugation, the supernatant was immediately detected by a microplate reader at 340 nm.

H_2_O_2_ has a characteristic absorption peak at 240 nm. CAT decomposes H_2_O_2_, so that the absorbance of the reaction solution at 240 nm decreases with the reaction time. CAT activity can be calculated according to the change rate of absorbance. Briefly, the algae cells were collected by centrifugation, and the extraction buffer in the kit was provided for ultrasonic crushing (power 200 W, work 3 s, interval 10 s, repeat 30 times). After centrifugation, the supernatant was immediately detected by a microplate reader at 240 nm.

MDA content and the activities of the four enzymes were calculated based on protein concentration. In this study, the BCA assay kit was used to determine protein concentration, and bovine serum albumin was used as the standard. The methods for measuring the expression levels of the genes encoding SOD, APX, GST, and CAT were consistent with those described in Section 2.4.

### 2.6. Analysis of Growth Performance and Photosynthetic Parameters

To monitor the growth of all strains, the OD values at a wavelength of 750 nm and the biomass of each algal strain were measured. The specific growth rate (*µ*, day^−1^) was calculated using the biomass and the following formula: (ln*N_t_* − ln*N_t_*_0_)/(*t* − *t*_0_), where *N_t_* and *N_t_*_0_ represent the biomass at times *t* and *t*_0_, respectively.

Considering the progress of microalgae stress tolerance research [22], to analyze the photosynthesis of all algal strains under high-temperature stress, the chlorophyll fluorescence parameters and photosynthetic O_2_ evolution rate were measured. In brief, 2 mL of algal cells was incubated in the dark for 15 min, and chlorophyll fluorescence parameters such as the maximum quantum yield of photosystem II (PSII) (Fv/Fm), the effective quantum yield of PSII [Y(II)], the relative electron transport rate (rETR), and the proportion of the closed PSII reaction center (1-qP) were monitored using a PAM fluorometer (Water-PAM, Walz, Nuremberg, Germany). The determination of photosynthetic O_2_ evolution rate was performed using a Chlorolab 2 + oxygen electrode system (Hansatech, King’s Lynn, UK). During the detection using the oxygen electrode, the light intensity was 100 μmol photons·m^−2^·s^−1^, and the temperature was 22 °C (normal culture group) and 30 °C (high-temperature culture group), respectively.

### 2.7. Statistical Analysis

All experiments in this study were performed triplicate, and data were presented as mean ± standard deviation (SD). Statistical analysis of the data was performed using GraphPad Prism 9.0 and SPSS 26.0. One-way ANOVA analysis and Tukey’s post hoc test were used to determine significant differences among algal strains.

## 3. Results

### 3.1. High Temperature Induces Upregulation of Nudix Expression

**Figure 1:**
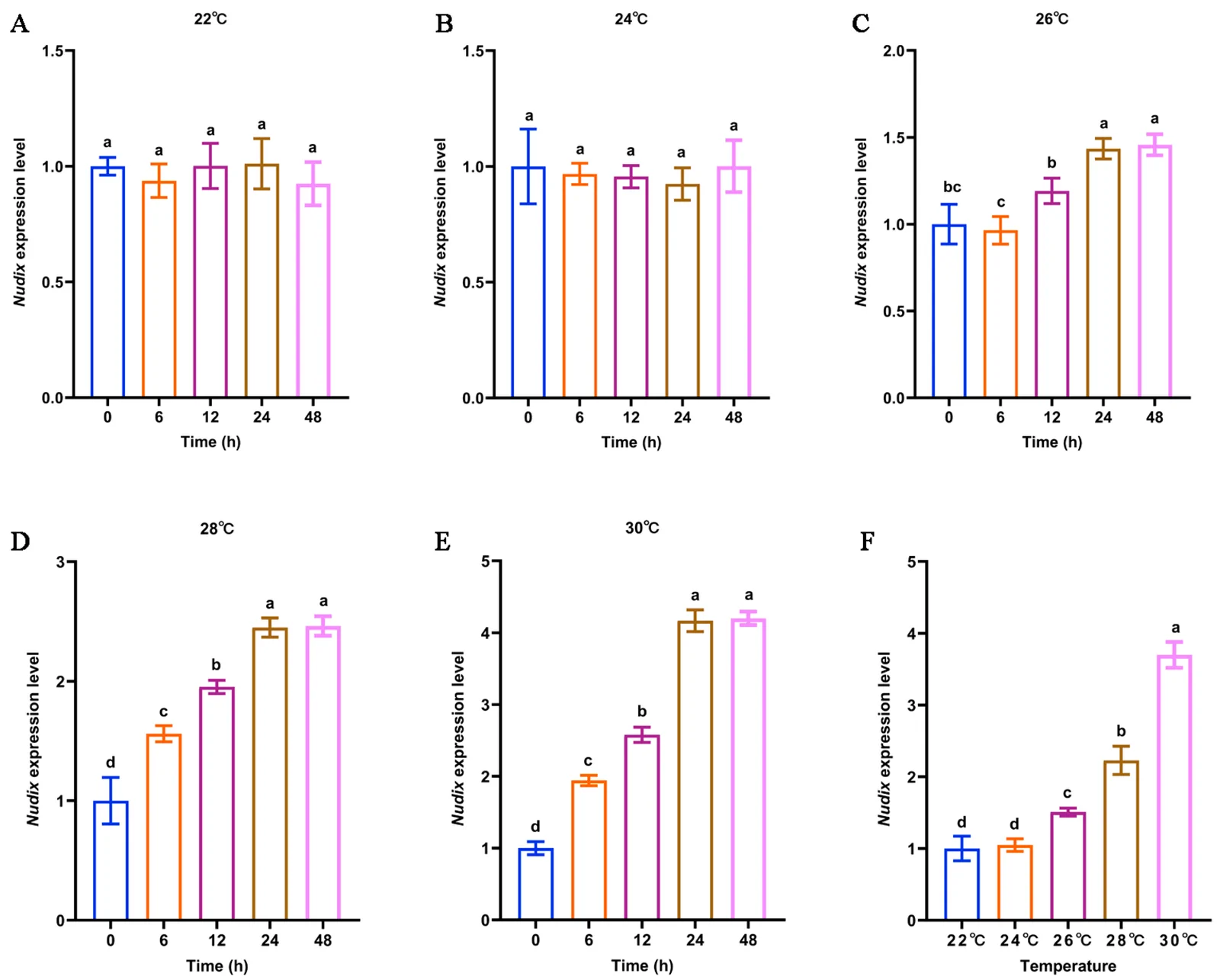
The expression levels of *Nudix* in *P. tricornutum* at (**A**) 22 °C; (**B**) 24 °C; (**C**) 26 °C; (**D**) 28 °C; (**E**) 30 °C. (**F**) *Nudix* expression levels after 48 h cultivation at different temperatures. The histone H4 gene served as the internal control. *n* = 3 biologically independent samples. The data were shown as the mean ± standard deviation (SD). Differences among groups were determined by one-way ANOVA and Tukey’s test. Values with different letters (a, b, c, d) indicate a significant difference between them (*p* < 0.05).

To explore whether Nudix is closely associated with diatom responses to high-temperature stress, we analyzed the expression levels of *Nudix* at different temperatures using the model diatom *P. tricornutum*. By qPCR, we found that no significant changes in *Nudix* expression levels were observed at 22 °C and 24 °C (Figure 1A,B). However, during the cultivation process, *Nudix* expression levels showed an upward trend under various high-temperature conditions (>26 °C) (Figure 1C–E). In particular, as the temperature increased, the extent of the upregulation in *Nudix* expression levels correspondingly increased (Figure 1F).

### 3.2. Detection of Nudix Subcellular Localization

**Figure 2:**
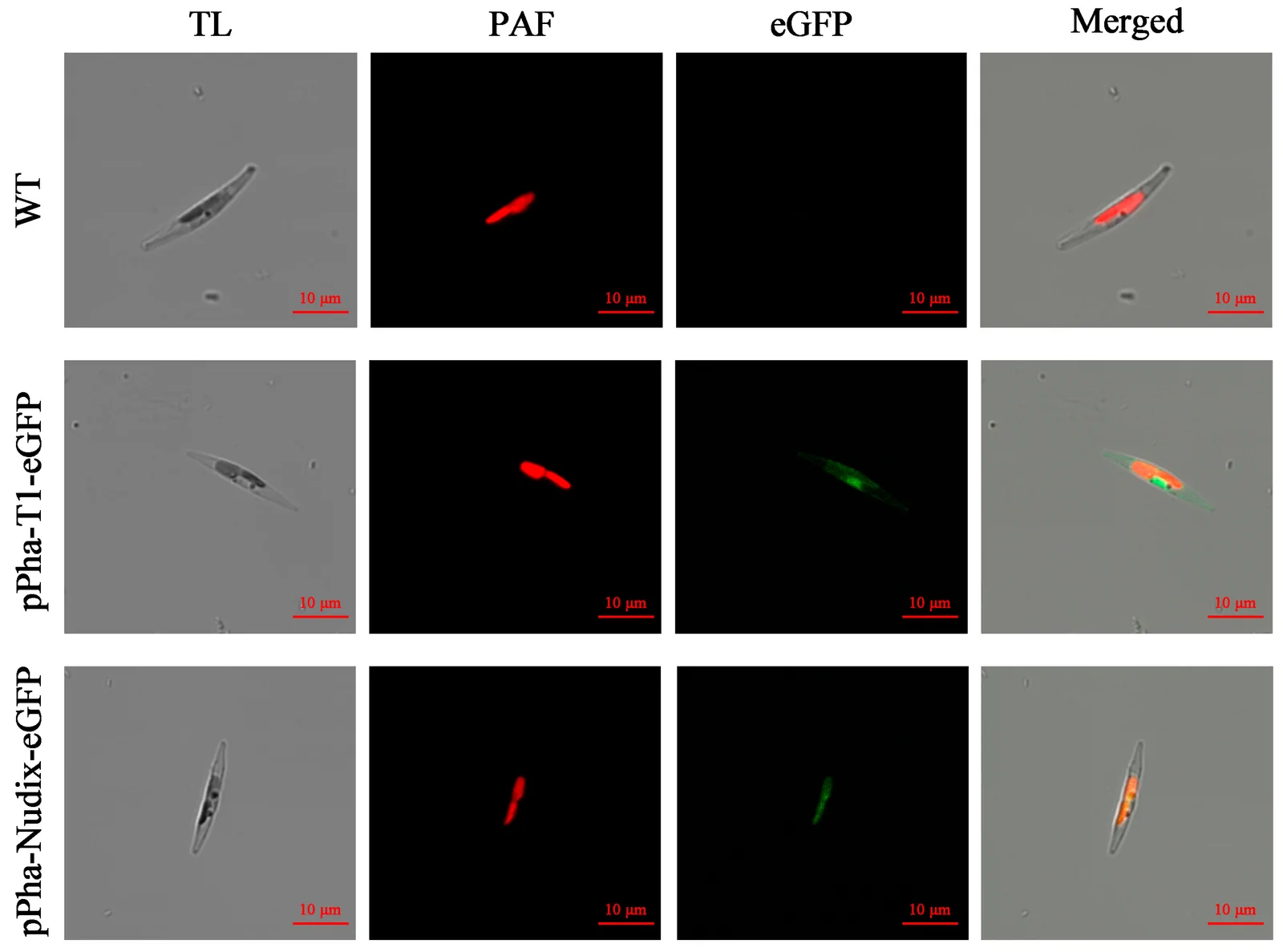
Subcellular localization of Nudix in *P. tricornutum*. WT: wild-type strain; pPha-T1-eGFP: transformation of eGFP alone; pPha-Nudix-eGFP: transformation of Nudix fused to eGFP; TL: transmitted light; PAF: plastid autofluorescence; eGFP: enhanced green fluorescent protein; Merged: overlay of plastid and green fluorescence. Scale bars in WT, pPha-T1-eGFP and pPha-Nudix-eGFP represent 10 μm.

To determine the subcellular localization of Nudix, we constructed a fusion expression vector containing Nudix with eGFP, and transformed it into algal cells. As shown in Figure 2, the green fluorescence signal of the control group (eGFP alone) was distributed throughout the algal cells. Compared with the control, the fusion of Nudix with eGFP resulted in the green fluorescence signals only located in chloroplast, and no green fluorescence was detected outside the chloroplast.

### 3.3. Generation of Nudix Overexpression and Silencing Strains

**Figure 3:**
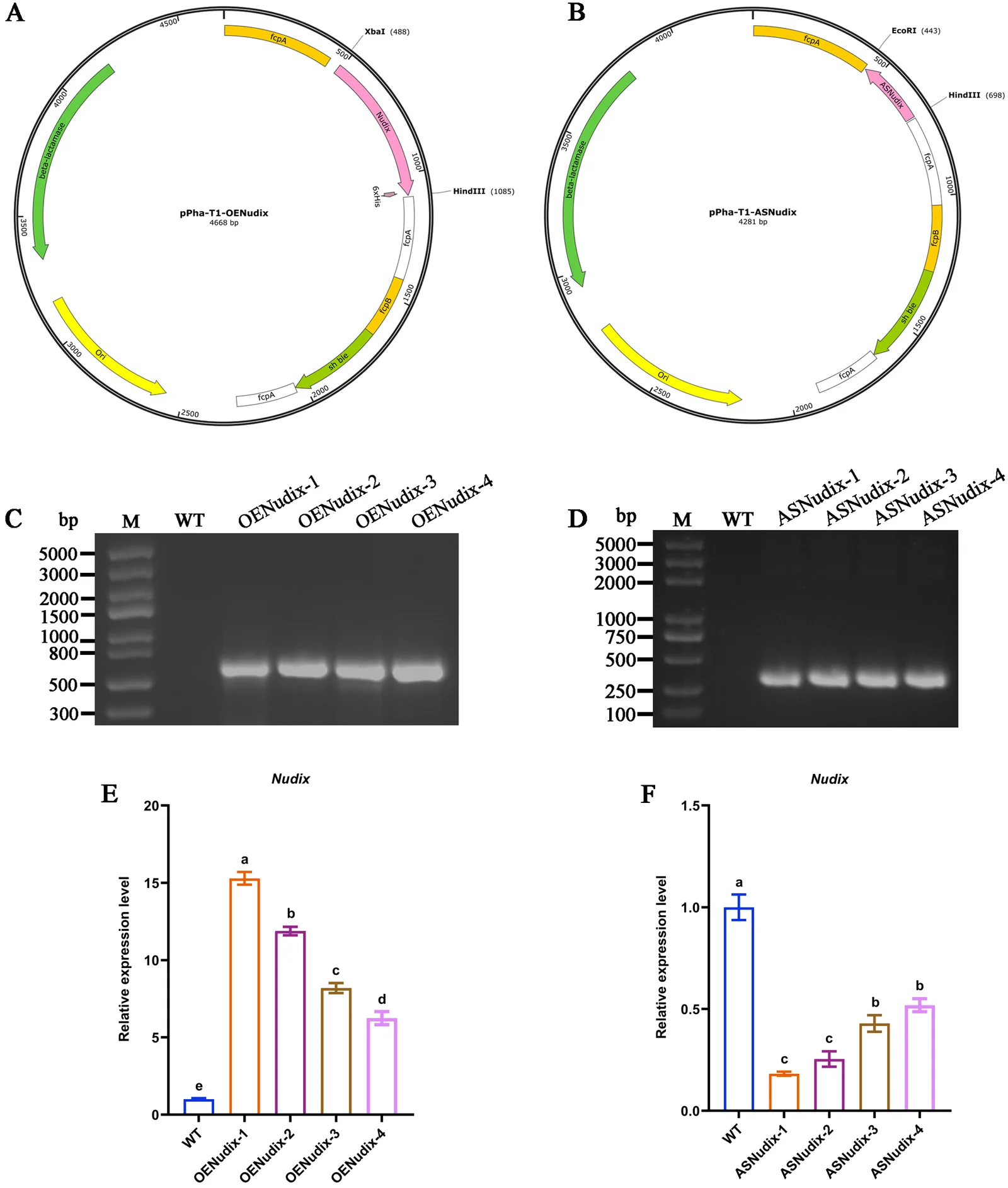
Generation of *Nudix* overexpression and silencing strains of *P. tricornutum*. (**A**,**B**) *Nudix* overexpression and silencing vector maps. (**C**,**D**) Preliminary molecular analysis of *Nudix* overexpression and silencing strains. (**E**,**F**) The expression levels of *Nudix* determined by qPCR. The histone H4 gene served as the internal control. WT represents wild-type strain; OENudix represents *Nudix* overexpression strain; ASNudix represents *Nudix* silencing strain. *n* = 3 biologically independent samples. The data were shown as the mean ± standard deviation (SD). Differences among groups were determined by one-way ANOVA and Tukey’s test. Values with different letters (a, b, c, d, e) indicate a significant difference between them (*p* < 0.05).

To investigate the function of Nudix in high-temperature responses, we generated *Nudix* overexpression and silencing strains using genetic engineering techniques. The integration of the overexpression and silencing vectors (Figure 3A,B) into the *P. tricornutum* genome was assessed by PCR. As shown in Figure 3C,D, corresponding specific amplified fragments were detected in the overexpression and silencing strains, respectively, but not in the WT strain. Next, the expression levels of *Nudix* in the overexpression and silencing strains were analyzed using qPCR. The expression levels of *Nudix* were significantly higher in overexpression strains and lower in silenced strains than in the WT strain (Figure 3E,F).

### 3.4. Nudix Improves High Temperature Stress Tolerance in P. tricornutum

**Figure 4:**
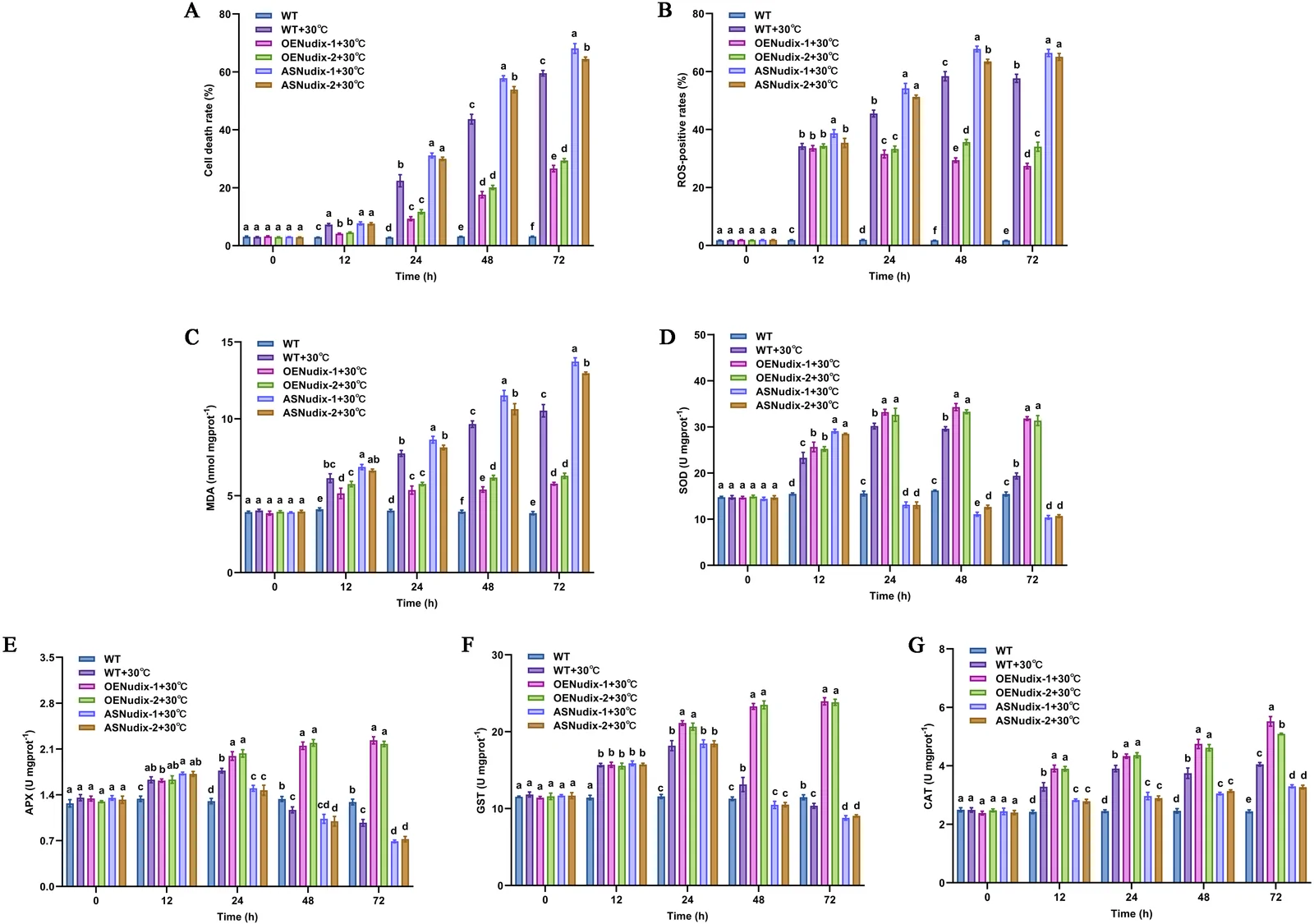
Effect of Nudix on the high-temperature tolerance of *P. tricornutum*. (**A**) Cell death rate. (**B**) ROS production. (**C**) MDA content. (**D**) SOD activity. (**E**) APX activity. (**F**) GST activity. (**G**) CAT activity. WT represents wild-type strain; OENudix represents *Nudix* overexpression strain; ASNudix represents *Nudix* silencing strain. *n* = 3 biologically independent samples. The data were shown as the mean ± standard deviation (SD). Differences among groups were determined by one-way ANOVA and Tukey’s test. Values with different letters (a, b, c, d, e, f) indicate a significant difference between them (*p* < 0.05).

To dissect the role of Nudix at high temperature, the high-temperature tolerance of *Nudix* overexpression and silencing strains under 30 °C was analyzed. As shown in Figure 4, compared with normal cultivation, high temperature resulted in serious oxidative damage in the WT strain; cell death rates (17.70-fold increase, *p* < 0.05), ROS (32.02-fold increase, *p* < 0.05) and MDA (1.72-fold increase, *p* < 0.05) were significantly increased, while antioxidant enzymes were activated (SOD activity improved by 25.33%, CAT activity improved by 65.76%, *p* < 0.05).

Under high-temperature stress, unlike the WT strain, the *Nudix* overexpression and silencing strains showed different high-temperature tolerance. As shown in Figure 4A, compared with WT, the cell death rate in the two overexpression strains was reduced by 55.24% and 50.53%, *p* < 0.05, while the cell death rate of those in the two silencing strains increased by 14.37% and 8.26%, *p* < 0.05. Compared with WT, ROS levels in the two overexpression strains were reduced by 52.46% and 40.92%, *p* < 0.05, while those in the two silencing strains increased by 15.17% and 12.83%, *p* < 0.05 (Figure 4B). MDA contents also showed results consistent with those of ROS levels: MDA in the overexpression strains decreased by up to 45.09%, *p* < 0.05, while those in the silencing algal strains increased by up to 30.28%, *p* < 0.05 (Figure 4C). In addition, compared with the WT strain, the antioxidant enzyme (SOD, APX, GST, CAT) activities of *Nudix* overexpression strain were significantly increased (*p* < 0.05), while the knockout strain activities were significantly reduced (*p* < 0.05) (Figure 4D–G).

**Figure 5:**
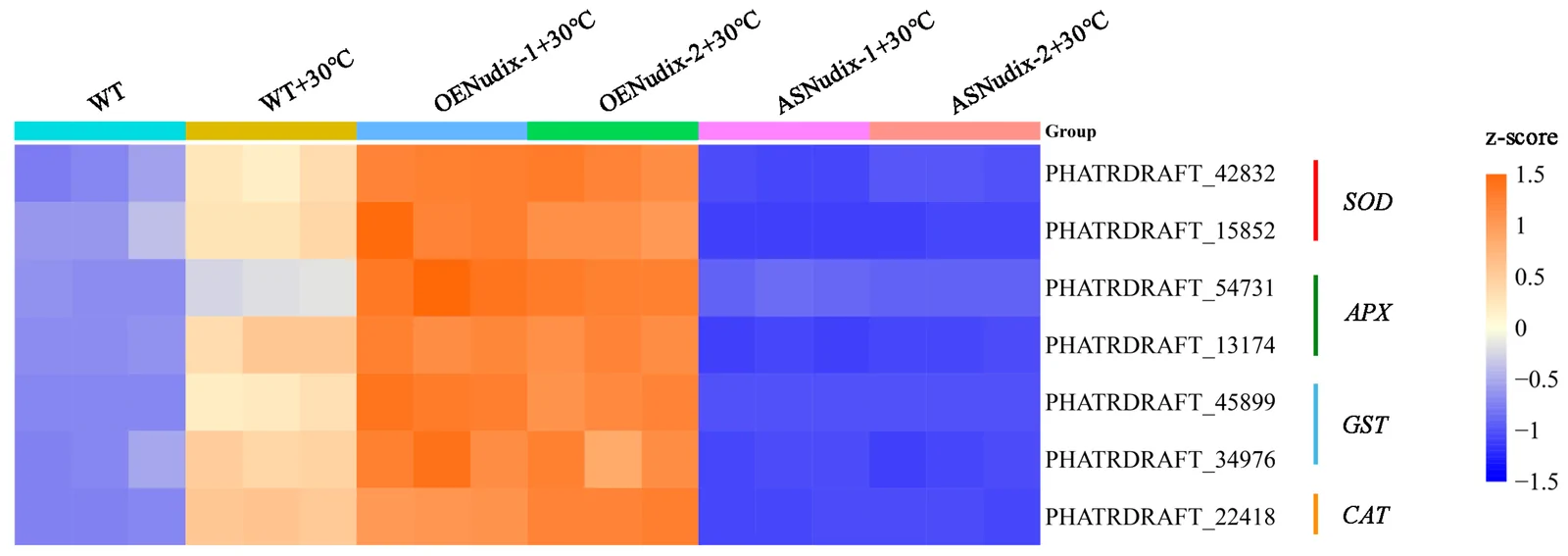
Effect of Nudix on the expression levels of the genes encoding SOD, APX, GST, and CAT of *P. tricornutum*. The histone H4 gene served as the internal control. WT represents wild-type strain; OENudix represents *Nudix* overexpression strain; ASNudix represents *Nudix* silencing strain. *n* = 3 biologically independent samples.

During further analysis of the expression levels of genes encoding antioxidant enzymes, as shown in Figure 5, we found that compared with WT, the two SOD-encoding genes were significantly upregulated in OENudix-1 by 61.30% and 60.02%, respectively, and in OENudix-2 by 60.00% and 45.38%, respectively (*p* < 0.05). Conversely, these two genes were significantly downregulated in ASNudix-1 and ASNudix-2, with expression levels being 82.52% and 84.97% lower in ASNudix-1 and 78.38% and 83.65% lower in ASNudix-2, respectively, compared with WT (*p* < 0.05). Compared with WT, the two APX-encoding genes were significantly upregulated in OENudix-1 by 157.14% and 33.41%, respectively, and in OENudix-2 by 145.16% and 30.92%, respectively (*p* < 0.05). Conversely, these two genes were significantly downregulated in ASNudix-1 and ASNudix-2, with expression levels being 66.65% and 73.65% lower in ASNudix-1 and 69.71% and 72.09% lower in ASNudix-2, respectively, compared with WT (*p* < 0.05). Compared with WT, the two GST-encoding genes were significantly upregulated in OENudix-1 by 76.10% and 34.43%, respectively, and in OENudix-2 by 64.30% and 27.32%, respectively (*p* < 0.05). Conversely, these two genes were significantly downregulated in ASNudix-1 and ASNudix-2, with expression levels being 91.12% and 64.24% lower in ASNudix-1 and 90.47% and 64.90% lower in ASNudix-2, respectively, compared with WT (*p* < 0.05). Compared with WT, the CAT-encoding gene was significantly upregulated in OENudix-1 by 27.80%, and in OENudix-2 by 39.38% (*p* < 0.05). Conversely, this gene was significantly downregulated in ASNudix-1 and ASNudix-2, with expression levels being 93.39% lower in ASNudix-1 and 93.02% lower in ASNudix-2 compared with WT (*p* < 0.05).

### 3.5. Nudix Enhances Photosynthetic Capacity and Growth Performance of P. tricornutum at High Temperature

**Figure 6:**
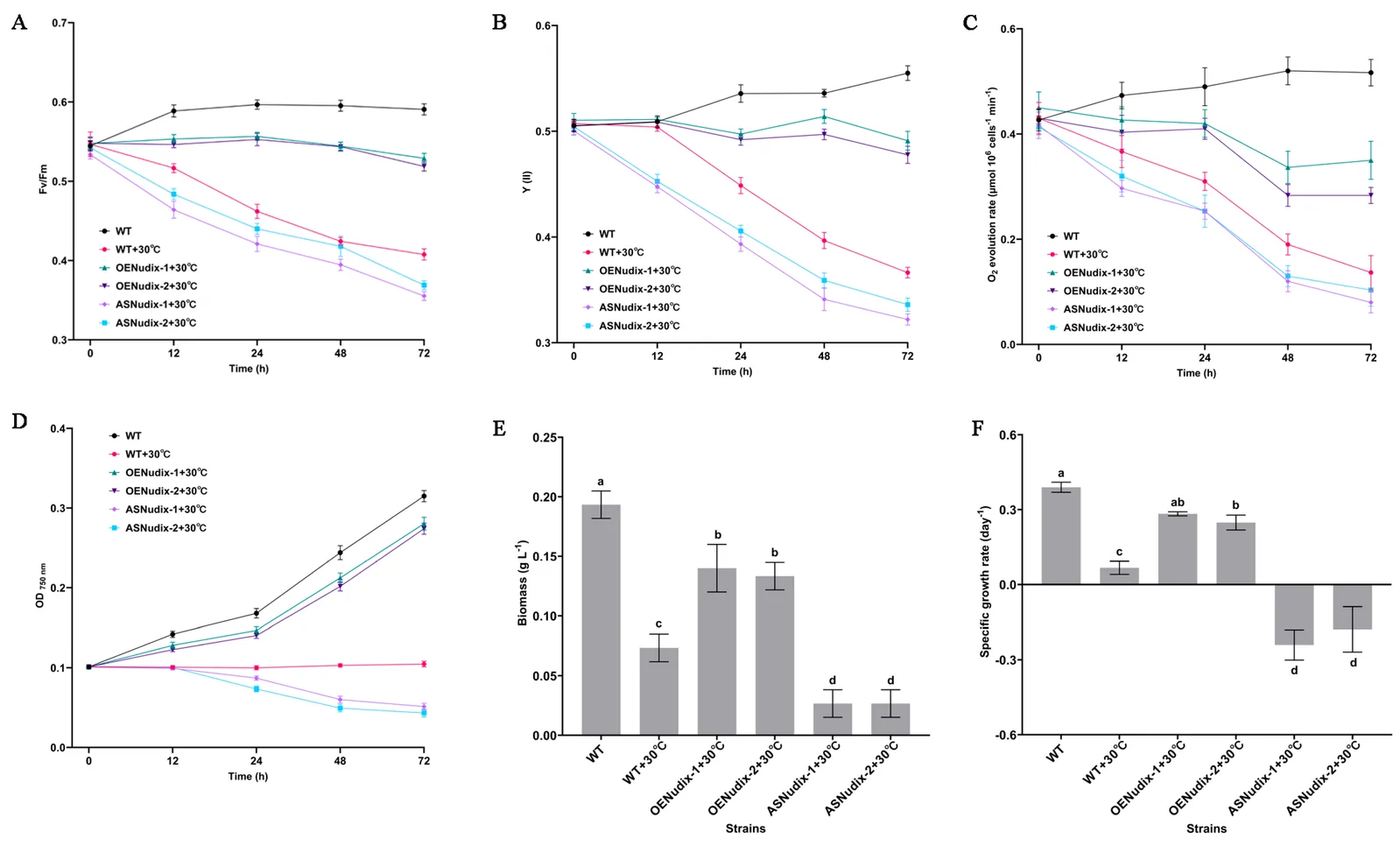
Effects of Nudix on the photosynthetic activity and growth of *P. tricornutum* under high temperature. (**A**) Fv/Fm. (**B**) Y(II). (**C**) Photosynthetic O_2_ evolution rate. (**D**) Growth curves. (**E**) Biomass. (**F**) Specific growth rate. WT represents wild-type strain; OENudix represents *Nudix* overexpression strain; ASNudix represents *Nudix* silencing strain. *n* = 3 biologically independent samples. The data were shown as the mean ± standard deviation (SD). Differences among groups were determined by one-way ANOVA and Tukey’s test. Values with different letters (a, b, c, d) indicate a significant difference between them (*p* < 0.05).

By analyzing photosynthesis under high temperature, we found that under high-temperature conditions, compared with the WT strain, *Nudix* overexpression strains exhibited significantly higher Fv/Fm, Y(II) and photosynthetic O_2_ evolution rate, whereas *Nudix* silencing strains showed significantly lower photosynthetic parameters (Figure 6A–C). In addition, we also analyzed the effect of Nudix on the growth of *P. tricornutum* under high-temperature (Figure 6D–F). Under high temperature stress, the growth performance of the *Nudix* overexpression strains was significantly better than that of the WT strain as photosynthesis enhanced, whereas the growth of the *Nudix* silencing strains was significantly worse than that of the WT strain as the photosynthesis decreased.

### 3.6. Nudix Inhibits ROS Production by Reducing the Excitation Pressure of the Photosynthetic Electron Transport Chain at High Temperature

**Figure 7:**
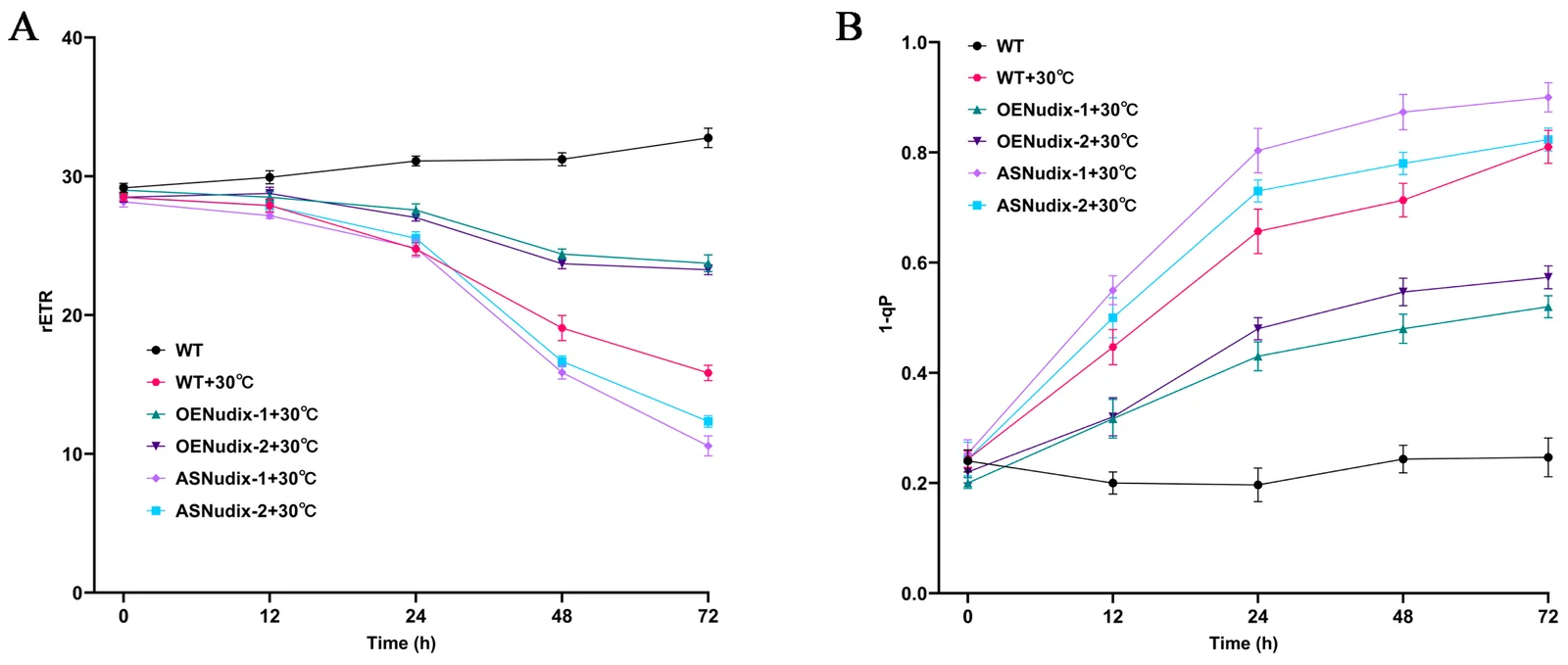
Effect of Nudix on the excitation pressure of the photosynthetic electron transport chain of *P. tricornutum* under high temperature. (**A**) rETP. (**B**) 1-qP. WT represents wild-type strain; OENudix represents *Nudix* overexpression strain; ASNudix represents *Nudix* silencing strain. *n* = 3 biologically independent samples. The data were shown as the mean ± standard deviation (SD).

Building on the finding that Nudix reduces the damage of high temperature to photosynthesis, this study further analyzed its effect on the excitation pressure of the photosynthetic electron transport chain. The results of photosynthetic electron transport showed that high temperature destroyed the photosynthetic electron transport process (significant reduction in rETR), and increased *Nudix* expression in the overexpression strains enhanced rETR, whereas reduced *Nudix* expression in the silencing strains exacerbated the inhibitory effect of high temperatures on rETR compared with that of WT strain (Figure 7A). Moreover, we analyzed the 1-qP content, a key parameter reflecting the redox state of the PQ pool of algal cells. As shown in Figure 7B, similar to rETR, high temperature significantly increased 1-qP in algal cells. Interestingly, *Nudix* overexpression reduces 1-qP; conversely, *Nudix* silencing strains increased 1-qP more than the WT strain.

## 4. Discussion

Diatoms, as the most diverse group of phytoplankton in the ocean, play a vital role in marine ecosystems [23]. In this study, we identified a chloroplast-localized Nudix hydrolase as a positive regulator of high-temperature tolerance in the marine model diatom *P. tricornutum*. Functional analyses showed that Nudix contributes to the maintenance of photosynthetic performance under high-temperature stress, thereby reducing the excitation pressure of the photosynthetic electron transport chain and limiting ROS accumulation. These findings suggest that Nudix is not merely a stress-responsive gene, but an active component of the thermal acclimation machinery in *P. tricornutum*.

Previous studies in higher plants have shown that Nudix hydrolases participate in stress responses by regulating cellular homeostasis, redox balance, and the turnover of potentially toxic metabolites [14,24,25,26,27]. However, no studies have yet reported whether Nudix is involved in the response to high-temperature stress. Our results extend the functional relevance of this family to marine diatoms and reveal a previously unrecognized role of Nudix in high-temperature adaptation. Notably, the chloroplast localization of Nudix suggests that its protective function may be closely associated with the stability of the photosynthetic apparatus.

To investigate the biological function of Nudix in high-temperature responses, we generated *Nudix* overexpression and silencing strains using genetic engineering techniques. Under high-temperature stress, increased expression of *Nudix* in the overexpression strains reduced ROS production and enhanced ROS scavenging capacity in algal cells, thereby decreasing lipid peroxidation and cell death rate to improve the heat tolerance of *P. tricornutum*. Conversely, once *Nudix* expression levels decrease, the ROS content of Nudix silencing strains was much higher than that of the WT, leading to reduced high-temperature tolerance and even massive algal cell death. Given growing evidence that environmental stress induces excessive accumulation of ROS and maintaining ROS homeostasis serves as the pivotal factor influencing high-temperature tolerance of photosynthetic organisms [28,29,30], combined with the results of this study, we analyzed that Nudix participates in the high-temperature response of *P. tricornutum* by regulating ROS levels.

Although Nudix can regulate ROS levels at high-temperature, how it affects ROS production and accumulation remains unclear. It is well known that temperature influences fundamental biological processes such as cell growth, photosynthesis, and respiration [31,32]. Among these, photosynthesis is one of the most heat-sensitive physiological processes in microalgae, and it is also the primary site of ROS production [23,32]. In alignment with this understanding, we analyzed photosynthesis-related parameters in algal strains with *Nudix* overexpression and silencing strains, and found that compared with the WT strain, the photosynthetic parameters of the *Nudix* overexpression strains were significantly increased at high temperature, while the silencing strains were significantly reduced. These results indicated that Nudix indeed maintains photosynthetic activity in algal cells under high temperature. Furthermore, subcellular localization results indicated that Nudix is a uniquely chloroplast-localized protein, which provides direct spatial evidence for its involvement in the regulation of photosynthetic activity under high temperature. To date, no studies have reported on the relationship between Nudix and photosynthesis in marine diatoms. Excitingly, a groundbreaking study on land plants revealed that members of the Nudix family play crucial roles in regulating photosynthetic pigment biosynthesis to support photosynthesis and growth [33], which complements the potential function of the Nudix family in photosynthesis and provides an important basis for this study. Thus, combined with the results of Nudix influencing the photosynthetic activity of *P. tricornutum* under high temperature stress in this study, we speculated that Nudix may influence ROS production by modulating photosynthesis.

More importantly, existing research has confirmed that under environmental stress, ROS production during photosynthesis is attributed to weakened photosynthetic activity and electron transport rates, resulting in the accumulation of energetic electrons in the photosynthetic electron transport chain, which in turn leads to the excessive reduction in the electron transport chain [22,34]. Our study further supports this view by measuring the photosynthetic electron transport rate and the redox state of the PQ pool. We found that under high temperature, Nudix enhances the photosynthetic electron transport rate in *P. tricornutum*, preventing energetic electron accumulation and excessive reduction in the photosynthetic electron transport chain, thereby suppressing ROS production; once Nudix is inhibited, the rate of electron transport slowed and the excitation pressure of the photosynthetic electron transport chain surged, resulting in excessive production of ROS. Together, these results confirmed that Nudix is a key regulatory hub for ROS production and high-temperature adaptation in *P. tricornutum*.

From a future perspective, understanding the molecular mechanisms underlying diatom high-temperature tolerance is essential for predicting how marine primary producers will respond to ongoing ocean warming. Diatoms play a central role in marine productivity and carbon cycling [3,4], and their ability to maintain photosynthetic activity under high temperature may strongly affect their ecological fitness in future oceans. By analyzing the function of Nudix and its underlying regulatory mechanisms, this study provides a potential molecular target for evaluating and improving thermal tolerance in diatoms. These findings not only broaden our understanding of Nudix function in marine photosynthetic organisms but also provide a foundation for future studies on diatom adaptation under climate change.

## 5. Conclusions

In summary, through the combination of genetic, biochemical and physiological approaches, this study identified Nudix as a critical regulator of high-temperature tolerance in *P. tricornutum* and showed the biological function of Nudix in high-temperature responses. Under high-temperature stress, Nudix mitigates the damage of high temperature to photosynthesis and reduces the excitation pressure of photosynthetic electron transport chain, thereby decreasing ROS production and improving the high-temperature tolerance of algal cells. These findings deepen our understanding of the mechanisms underlying diatom high-temperature adaptation. It should be noted that this study performed the functional analysis of Nudix, and its specific regulatory mechanisms remain unclear. Future efforts should prioritize the investigating of the molecular mechanisms by which Nudix regulates photosynthesis and high-temperature tolerance in diatoms.

## Data Availability

The original contributions presented in this study are included in the article/Supplementary Material. Further inquiries can be directed to the corresponding authors.

## Supplementary Materials

The following supporting information can be downloaded at: https://www.mdpi.com/article/10.3390/microorganisms14081625/s1, Figure S1: The expression level of *Nudix* in *P. tricornutum* with significant differences in high temperature tolerance; Figure S2. The gating strategy according to FSC/SSC of *P. tricornutum*; Table S1: List of primers used in this study.
