# Supplementary material for: Nudix Hydrolase as a Target for Regulating High Temperature Tolerance in Marine Diatoms
Source: Microorganisms. 2026 Jul 25;14(8):1625. doi: 10.3390/microorganisms14081625 (PMC13515168; doi:10.3390/microorganisms14081625)
Supplement: Supplementary file 1 [file microorganisms-14-01625-s001.zip › microorganisms-4428672-supplementary.pdf]

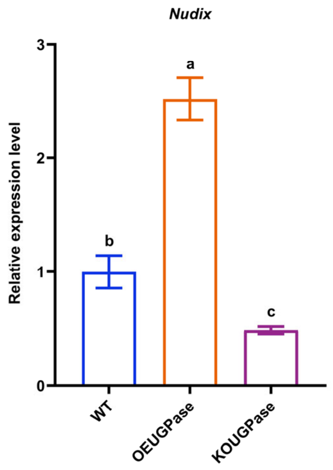

Figure S1. The expression level of *Nudix* in *P. tricornutum* with significant differences in high temperature tolerance. The new data presented in this figure are part of this study. The histone H4 gene served as the internal control. WT represents wild-type strain; OEUGPase represents *UGPase* overexpression strain; KOUGPase represents *UGPase* knockout strain. $n = 3$ biologically independent samples. The data were shown as the mean ± standard deviation (SD). Differences among groups were determined by one-way ANOVA and Tukey's test. Values with different letters (a, b, c) indicate a significant difference between them ($P < 0.05$).

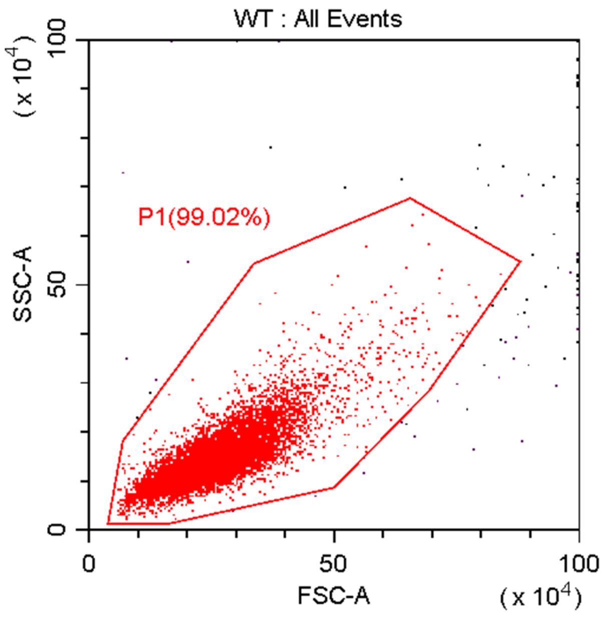

Figure S2. The gating strategy according to FSC/SSC of *P. tricornutum*.

Table S1. List of primers used in this study.

Gene number	Primer name	Primer sequence (5'→3')	Purpose
PHATRDRAFT_31704	OENudix-f	TGTTGCTTCGTCGGGAATCCCTAC	Identification of strains
	OENudix-r	TCGGTCAACGCCATGTCTTC	Identification of strains
PHATRDRAFT_31704	ASNudix-f	TGTTGCTTCGTCGGGAATCCCTAC	Identification of strains
	ASNudix-r	TTCCGCGGAAGCAGTGATGT	Identification of strains
PHATRDRAFT_31704	Nudix-f	GAGCTCAAAATCATGCGACCAA	qPCR
	Nudix -r	GTCAACGCCATGTCTTCCAAAA	qPCR
PHATRDRAFT_42832	SOD1-f	CGTTCTTCGAAAATCCCCACAG	qPCR
	SOD1-r	GGGAGGCGTAGTCGTAAGTAAG	qPCR
PHATRDRAFT_15852	SOD2-f	GTGGGTGACTTGGGAAACATTG	qPCR
	SOD2-r	CCAGAACAGAATGAGGACCCAA	qPCR
PHATRDRAFT_54731	APX1-f	GACGAATCGATTCAAACGGTCC	qPCR
	APX1-r	GACTTTACGTAAATGACCGGCG	qPCR
PHATRDRAFT_13174	APX2-f	CCGATATGGCACTCATTCAGGA	qPCR
	APX2-r	GCAAAAGCTTCGGCAAAATCAG	qPCR
PHATRDRAFT_45899	GST1-f	AATCACTCGTGGCGATCGATTA	qPCR
	GST1-r	CAGGGTTGCAAAATGTTCTCGT	qPCR
PHATRDRAFT_34976	GST2-f	TAATAGCTTCCTTACGGGCGAC	qPCR
	GST2-r	GTTCATGATGTCGGCTGTCTTG	qPCR

PHATRDRAFT_22418	CAT-f	GCGGTCGACATTAATCCCTTTG	qPCR
	CAT-r	GTCTTTCGGATTGCGATTCAGG	qPCR
PHATRDRAFT_34971	H4-f	GTGGTAAAGGAGGCAAGGGTC	qPCR
	H4-r	GGCGTGCTCGGTATAGGTGA	qPCR
